# Self-tuition as an essential design feature of the brain

**DOI:** 10.1098/rstb.2020.0530

**Published:** 2022-02-14

**Authors:** David A. Leopold, Bruno B. Averbeck

**Affiliations:** ^1^ Section on Cognitive Neurophysiology and Imaging, Laboratory of Neuropsychology, National Institute of Mental Health, National Institutes of Health, Bethesda, MD, USA; ^2^ Neurophysiology Imaging Facility, National Institute of Mental Health, National Institute of Neurological Disorders and Stroke, National Eye Institute, National Institutes of Health, Bethesda, MD, USA; ^3^ Section on Learning and Decision Making, Laboratory of Neuropsychology, National Institute of Mental Health, National Institutes of Health, Bethesda, MD, USA

**Keywords:** neurodevelopment, hypothalamus, telencephalon, prosomeric model, epigenetic

## Abstract

We are curious by nature, particularly when young. Evolution has endowed our brain with an inbuilt obligation to educate itself. In this perspectives article, we posit that self-tuition is an evolved principle of vertebrate brain design that is reflected in its basic architecture and critical for its normal development. Self-tuition involves coordination between functionally distinct components of the brain, with one set of areas motivating exploration that leads to the experiences that train another set. We review key hypothalamic and telencephalic structures involved in this interplay, including their anatomical connections and placement within the segmental architecture of conserved forebrain circuits. We discuss the nature of educative behaviours motivated by the hypothalamus, innate stimulus biases, the relationship to survival in early life, and mechanisms by which telencephalic areas gradually accumulate knowledge. We argue that this aspect of brain function is of paramount importance for systems neuroscience, as it confers neural specialization and allows animals to attain far more sophisticated behaviours than would be possible through genetic mechanisms alone. Self-tuition is of particular importance in humans and other primates, whose large brains and complex social cognition rely critically on experience-based learning during a protracted childhood period.

This article is part of the theme issue ‘Systems neuroscience through the lens of evolutionary theory’.

## 1. Introduction

A critical early mission of the brain is to direct its own education [1]. Genetic programmes are limited in their capacity to confer sophisticated behaviours. However, the brain has evolved systems that develop specialization through experience. Thus, acquiring experience is itself part of a developmental programme that allows inherently adaptable neural circuits to reach their normal functional maturity and behavioural capacities. In humans and other primates with large brains and long childhoods, neural specialization comes about gradually over years of experience. Across species, some learned specializations are shared broadly, whereas others are linked to a given ecology or sensory modality.

In this perspectives article, we posit that the vertebrate forebrain has evolved to support an interplay between brain areas that drives its own education based on a curiosity-driven exploration of the environment. We refer to this process as *self-tuition.* We begin by describing a broad conceptual framework for understanding this behavioural process. We propose that developmental self-tuition follows from an innate motivation to explore one's environment in a targeted way. After laying out our proposal, we review key neural structures and anatomical brain architecture that may be involved in self-tuition. We focus on the hypothalamus and its generation of motivated behaviours during early life. These behaviours are commonly associated with homeostasis and basic survival [2,3]. We propose that, in addition, these behaviours and the resulting experiences are critical drivers for the normal development of the telencephalon. We then provide examples of how early life behaviours can serve the dual role of immediate survival and longer-term training of the brain. We highlight one particularly important mode of statistical learning related to developing expertise in face recognition in primates. While we posit that this aspect of brain operation is a conserved feature of the vertebrate brain, we focus on mammals, and particularly primates, whose long childhood affords them an extended developmental window for this process to unfold.

## 2. The brain's obligation to educate itself

It is well established that neural circuits throughout the brain become specialized based upon an animal's behavioural experiences with the environment, and that this learning is most intense during development (for a recent review, see [4]). The behavioural and neural aspects of these processes are well understood in, for example, the early visual system, where spontaneous eye opening, and the subsequent exposure to visual input during a critical period of plasticity, is required for the normal development and maturation of its circuits [5]. We posit that such preordained coordination between spontaneous behaviours and developmental plasticity is a central principle of brain design that shapes its specialization for a wide range of behaviours. For example, social circuits in the brain are trained through innate but complex behaviours such as play, conflict, and, in the case of primates, preoccupation with important visual stimuli such as faces. Just as eye opening is required for normal development of the early visual system, early life exploratory and curiosity-driven behaviours are critical for the normal development of brain regions invested in other cognitive operations.

The topic of developmental plasticity often comes to the fore when scientists grapple with neuroscientific problems that feature the complex interaction between genetics and environment [6–11]. Less often discussed, however, is the brain's critical and evolved obligation to educate itself through the active solicitation of species-appropriate interactions with the environment. Instead of pre-programming an extensive array of environmental knowledge, the brain has evolved to motivate certain types of exploration of the environment. This exploration leads to its learning of statistical regularities and gradual acquisition of knowledge and expertise within its neural systems, which then support the adult repertoire of behaviours.

**Figure 1.**
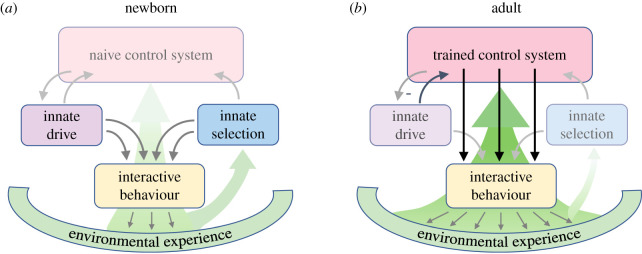
Theoretical depiction of the expression and development of self-tuition in the brain. (*a*) In the newborn brain, interactive behaviour with the environment is strongly driven by innate factors, including the internal generation of motivated behavioural states (purple, e.g. in the hypothalamus) and the biased processing of important stimuli (blue, e.g. in sensory pathways). The resulting experiences lead to gradual functional specialization of adaptable control circuits (pink, e.g. in the telencephalon). (*b*) Following extensive developmental experience, the adult brain engages in a broader range of interactive behaviours, which are orchestrated more directly by the trained central control circuits. Innate drives and biases continue to promote various behaviours in the adult, but these can now be overridden by the trained control circuits.

Programmed self-tuition is thus a recursive and dynamic ingredient in the normal development of the brain. It is schematized in figure 1. A newborn animal enters the world with a limited behavioural repertoire that includes some innate motivational drives and sensory selection biases. Initially, vast portions of the brain consist of naive but inherently adaptable circuits. Ultimately, these adaptable circuits are destined to gain control over the tactical aspects of everyday behaviour (figure 1*a*). However, in this nascent stage, behavioural interactions with the outside world operate within a relatively narrow range, often within a protected maternal environment. As the animal first experiences the consequences of its own actions and interactions with the environment, circuits throughout the brain begin to incorporate expertise about the details of sensory stimuli and events, motor execution, and cognitive and social behaviours. As the animal grows, its experiences broaden. The adaptable elements of the brain become increasingly specialized and autonomous, leading to a gradual shift in the basic operational principles of the brain (figure 1*b*). Over time, trained circuits begin to subsume control over motivated behaviour, incorporating their learned expertise in various cognitive domains to add sophistication and contingency to the behavioural repertoire. A more refined curiosity continues to promote environmental interactions that foster new modes of self-tuition, as some innate drives and stimulus biases continue to prod the adult brain to take certain actions. In the vertebrate brain, the key players in early life self-tuition are the hypothalamus, as the tutor, and the telencephalon, as the student that gradually becomes the master. The hypothalamus can play the dual role of motivating exploratory behaviour, and providing feedback, via interactions with the dopamine system, when a behaviour has achieved a goal that satisfies a need [12–15].

The hypothalamus is known best for initiating behavioural drives and controlling the neuroendocrine system. Its structure and development are highly conserved among vertebrates [16,17]. The selective stimulation of hypothalamic circuits in the adult brain initiates a range of natural behaviours that are aimed to satisfy physiological and reproductive needs [2,18–23]. Hypothalamic activity is critical for survival, both of the individual and of the species [24,25], through eating, drinking, thermoregulation, defensive behaviours, reproduction and rearing of young [2]. Many of these survival behaviours have additional consequences, emphasized here, of gradually shaping and specializing adaptable portions of the brain, most notably in the telencephalon. While knowledge of the ontological onset and progression of specific hypothalamic circuits driving such behaviours is imprecise, these circuits are likely to be most important early in life [1], when exploration is most advantageous for adaptive systems [26,27]. Whether such behaviours should be called ‘motivated’ in a traditional sense, may be a semantic matter. In his drive reduction theory, Hull suggested that motivation follows from increased drives or physiological needs, and that satisfaction of these needs is itself reinforcing [28]. This view of reinforcement, and the behaviour it motivates, is more closely related to survival than abstract behavioural concepts like reward and punishment [29,30]. This broader conception of reinforcement covers open loop motivational schemes such as those at play in spontaneous behaviours. Thus, reinforcement of hypothalamus-driven early life behaviours can be seen to be manifest over both short and long timescales.

The telencephalon, by contrast, is an instrument for developing expertise and learning complex behaviours. In the early postnatal period, as an animal engages interactively with the environment, the telencephalon is subject to continual modification as animals are exposed to important features of their environment. While its basic components and anatomical connections are in place at the time of birth in primates [31–38], abundant neural plasticity persists during the postnatal period. Dendritic spines and synapses proliferate after birth [39,40], peaking in different brain areas in the first months or early years of life before declining to adult levels during adolescence [41]. Exuberant white matter projections undergo pruning, including long-range descending projections [42,43]. Postnatal neurogenesis is thought to support forms of early life plasticity in the telencephalon [44–46] and cerebellum [47]. Patterns of myelination and gene expression related to cell differentiation continue to evolve gradually, through adolescence [48,49]. Within the cerebral cortex, one view holds that the most delayed maturation and protracted plasticity takes place in high-level associative neocortical areas [39,50–58]. A contrasting view suggests that the limbic areas retain the highest plasticity in the adult cortex, maintaining higher expression of markers for axonal growth and lower levels of myelin and perineuronal nets [59,60].

The steady stream of behavioural interactions initiated by the hypothalamus during early life, together with the multiple forms of plasticity expressed by the telencephalon during that period, are the necessary ingredients for self-tuition. In the next section, we go deeper into the anatomical architecture of the vertebrate forebrain, discussing key aspects of the telencephalic and hypothalamic circuitry. We highlight the shared segmental relationship of these two structures, as well as the patterns of connections that shape their cooperative role in the brain's self-tuition.

## 3. The hypothalamus and telencephalon in context

### (a) Segmental layout of the vertebrate brain

**Figure 2.**
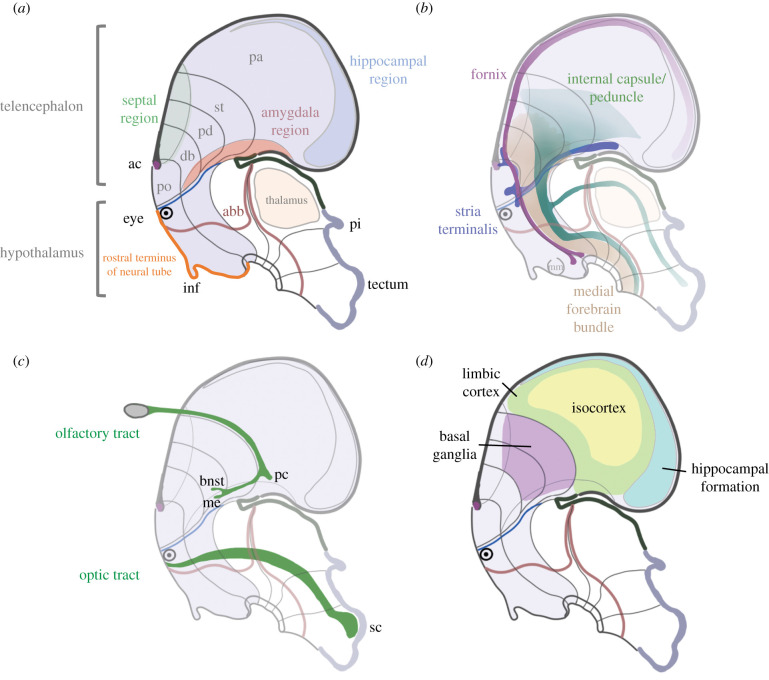
Neural pathways important for self-tuition in the context of the prosomeric model. (*a*) Depiction emphasizing the dorsal position of the telencephalon relative to the hypothalamus, with the acroterminal region of the hypothalamus (orange) identified as the rostral margin of the neural tube. Within the schematic of the flattened telencephalic vescicle, the septal and amygdala regions are shown to span subpallial and pallial territories and the hippocampal territory is identified at the pallial margin, corresponding to the medial pallial terrirtory. Adapted from Puelles *et al.* and Puelles & Rubenstein [62,63]. (*b*) Depiction of four prominent white matter tracts that facilitate communication between the hypothalamus and telencephalon. (*c*) Entry of olfactory and visual pathways into the brain through the telencephalon and hypothalamus, respectively, including primary targets where important stimuli can be identified through innate mechanisms. (*d*) Classic naming of telencaphalic components that participate in different types of learning, including early life learning. (abb, alar-basal boundary; ac, anterior commissure; bnst, bed nucleus of the stria terminalis; db, diagonal band; inf, infundibulum; me, medial nucleus of the amygdala; mm, mamillary bodies; pa, pallium; pc, piriform cortex; pd, pallidum; pi, pineal gland; po, preoptic area; sc, superior colliculus; st, striatum.).

The basic layout of the vertebrate brain has been conserved over roughly half a billion years [61]. In the past decades, a systematic mapping of its genoarchitecture during development has transformed our understanding of its structural components and their segmental relationships. At the heart of the issue, which is captured in the prosomeric model of brain organization [62,63] (figure 2), is the anatomical relationship between the hypothalamus and telencephalon within the neural tube. As these two critical elements are also at the core of developmental self-tuition, it is worth describing briefly the prosomeric model, the positioning of these key structures, and how this new framework bears on our conception of other relevant pathways, such as prominent white matter tracts and sensory pathways.

The prosomeric model is a segmental perspective that overturns a century-old view on the layout of the vertebrate forebrain. Most relevant for this article, the classical depictions of the neural tube layout considered the hypothalamus to be a portion of the diencephalon, and therefore to lie caudal to the telencephalon in a separate segment of the brain. However, myriad studies of comparative genoarchitecture gathered in the last two decades now place the hypothalamus at the rostral-most portion of the neural tube, with the classical telencephalon (end-brain) identified as its dorsal elaboration [63,64] (figure 2*a*). As a consequence, the hypothalamus and traditional telencephalon are now believed to occupy the same segmental territory of the neural tube (neuromere). Within this neuromere, the telencephalon bears a similar anatomical relationship to the hypothalamus as the tectum bears to the midbrain or the cerebellum bears to the rostral pons. The fact that both structures reside within the same neuromere may be important from an evolutionary perspective. Structures occupying the same brain segment are more likely to retain their basic organization and interrelationship through evolutionary change [65]. In the case of developmental self-tuition, this conserved organization may translate to functional principles, namely the coordinated and progressing early life roles of the hypothalamus and telencephalon.

The prosomeric framework alters not only the topological view of brain areas, but also their interconnections. The connections in and out of the telencephalon are particularly relevant to the present article (figure 2*b*), and are described more fully in the next section. In reference to the brain's segmental structure, some important fibre pathways such as the fornix, connecting hippocampal circuitry to the septum and mammillary bodies, remain largely within the same brain segment. Other fibre pathways such as corticothalamic/corticotectal pathways, providing cortical input to alar aspects of the diencephalon and midbrain, turn to run longitudinally and thus cross between brain segments. Likewise, sensory pathways that enter the central nervous system pass through different neuromeric territories and can be viewed in the context of the prosomeric model. This is shown for the two senses featured in this article (figure 2*c*). Visual fibres enter the hypothalamic tissue, with the bulk of projections passing through multiple neuromeres to its caudalmost destination in the optic tectum. Olfaction, by contrast, enters through the olfactory bulb, which is part of the telencephalon, and projects principally to areas within the telencephalon. Finally, within the telencephalon, pallial structures (e.g. isocortex, limbic cortex, hippocampus, olfactory cortex) have a concentric arrangement [59,66] (figure 2*d*).

This emerging view of forebrain organization thus places the tutor (i.e. the hypothalamus) in the same conserved brain segment as the student (i.e. the telencephalon). In the simplest formulation, there would need to be no direct interaction between these structures. The hypothalamus could train the telencephalon only through the generation of behaviour, and the telencephalon would benefit from this experience. However, the evidence suggests that the interconnections between these two structures makes the process of self-tuition more complicated, and more interactive, than that simple model. In the next section, we review several important projections between the hypothalamus and the telencephalon. The common denominator of these connections is that they articulate motivational circuits, giving the hypothalamus influence over telencephalic actions.

### (b) Pathways for hypothalamic influence over the telencephalon

The hypothalamus can initiate behaviour through many pathways. It exerts substantial control over the endocrine system, regulating hormonal activity that can influence early life behaviour in all vertebrate species [3]. It can also stimulate directly motor and autonomic centres through its descending projections to the brainstem and spinal cord [42,67]. Anatomically, the layout of the hypothalamus [68], its input and output projections [69], intrinsic connectivity [70], relationship to the endocrine system [71] and internal functional differentiation are daunting and difficult to summarize [2].

**Figure 3.**
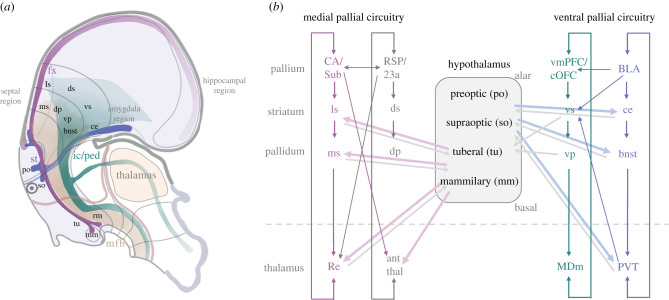
Hypothalamic pathways for engaging the telencephalon in complex behaviours. (*a*) Pathways and relevant hypothalamic, pallial and subpallial territories depicted in the context of the prosomeric model. (*b*) Subset of known connections between hypothalamus and limbic thalamocortical circuits. Each of four such circuits is depicted as loops, following the conventions of Alexander *et al.* [72]. The hypothalamus projects to different medial pallial (i.e. hippocampal) and ventral pallial (e.g. amygdala) circuitry in a coarsely topographic manner. For clarity, neuromodulatory connections, such as those stemming from peptidergic neurons (orexin, oxytocin, vasopressin) are omitted from this diagram. (ant thal, anterior thalamus; BLA, basolateral portion of the amygdala; bnst, bed nucleus of the stria terminalis; CA, cornu ammonis territory of the hippocampus; ce, central nucleus of the amygdala; cOFC, caudal portion of the orbitofrontal cortex; dp, dorsal pallidum; ds, dorsal striatum; fx, fornix; ic/ped, internal capsule/peduncle; mfb, medial forebrain bundle; MDm, medial portion of the medial dorsal nucleus of the thalamus; mm, mamillary bodies; ms, medial septum; ls, lateral septum; po, preoptic area; PVT, paraventricular nucleus of the thalamus; rm, retromamillary area; RSP, restrosplenial cortex; so, supraoptic area; st, stria terminalis; tu, tuberal region; Sub, subiculum; vmPFC, ventromedial portion of the prefrontal cortex; vp, ventral pallidum; vs, ventral striatum.).

Here we review one interesting set of connections, which may be relevant for controlling complex behaviours during early life. These are the anatomical projections into telencephalic circuitry. The principal motif of these projections (figure 3) is innervation of subpallial structures in limbic areas, namely the septal nuclei and extended amygdala. Such connections are considered part of the limbic brain and have long been recognized as important for motivation [73–75]. In the context of early life self-tuition, such projections afford the hypothalamus some control over the expertise of the telencephalon to carry out goal directed behaviour. Figure 3 presents several important hypothalamic projections to telencephalic circuitry, which is presented in the context of parallel recurrent pathways involving pallial, striatal, pallidal and thalamic structures [24,72].

The direct influence of the hypothalamus comes through three predominant pathways [69]. The first hypothalamus pathway projects to subpallial structures, including bed nucleus of the stria terminalis (bnst) and septum, which interact principally with the amygdala and hippocampal circuitry, respectively [24]. These pathways are perhaps the best example of the hypothalamic influence on telencephalic motivational circuits, perhaps influencing the expression of exploratory behaviours governed by associated pallial structures such as the amygdala and hippocampus, respectively [76,77].

The second hypothalamic pathway projects to thalamic nuclei that themselves project to the telencephalon. Interestingly, this relay in the thalamus may be analogous, with respect to its circuit organization, to sensory relay pathways [78]. The recipient thalamic structures include the paraventricular nucleus (PVT), and the anterior thalamic and reuniens nuclei, which in turn project to the amygdala and hippocampal associated circuitry, respectively [69]. The hypothalamic–thalamic projections exhibit a coarse topography, with alar hypothalamic regions projecting primarily to the PVT [79], and basal regions (primarily the mammillary bodies) projecting to the nucleus reuniens and the anterior thalamic nuclei [80]. This topography may reflect different hypothalamic signals impinging on different circuits. For example, it has been suggested that the alar (previously rostral) hypothalamus establishes behavioural goals including approach to food, water, a sexual partner, or escape from a predator, whereas the basal (previously caudal) hypothalamus, particularly the mammillary nuclei, is important for orienting, locomotion and navigation [24].

The third hypothalamic pathway is composed of direct projections to the telencephalon, including its pallial components. These projections are notably from neuromodulatory populations that deliver neuropeptides such as orexin, oxytocin, vasopressin and histamines [81–85]. This set of projections is known to have a significant impact on behaviour, including complex social behaviours, and is likely to play an important role in early life tuition.

Thus, in addition to its direct control of motor circuits through descending projections [24], the hypothalamus has the ability to intervene directly in the expression of behaviour initiated by the telencephalon. This intervention is principally through telencephalic motivational centres within limbic areas. These multiple pathways afford the hypothalamus multiple layers of control over complex behaviour, which may be particularly strong during early life but also persist into adulthood. In the adult, they have the potential to weigh in on, and sometimes override, important aspects of cognition and decision-making [3]. In the next section we explore expression of early life motivated behaviour, the way it is shaped by innate stimulus preferences, and principles by which the telencephalon can learn from the resulting experiences.

## 4. The functional expression of self-tuition

In the above sections, we laid out theoretical considerations and anatomical features of the vertebrate brain thought to contribute to the generation of early life behaviours. Here, we discuss how a few such behaviours are manifest in mammals, often serving dual roles of immediate survival and long-term training of the telencephalon and shaping of behaviour.

### (a) Prenatal behaviours

Some behaviours that can lead to experience-based learning are initiated *in utero*. Thus, some modes of self-tuition can begin before an animal is even born. This is particularly evident in precocial animals such as ungulates [86,87] and guinea pigs [88], whose ecology requires their locomotion shortly after birth. However, even extremely altricial species exhibit *in utero* behaviours, such as the wallaby, whose postnatal climbing movements that are required to ascend to the pouch and milk supply, first appear days before emerging from the birth canal [89]. In humans and other primates, *in utero* motor programmes prompt the fetus to move its body spontaneously, including the limbs, fingers, mouth and eyes [90–92]. Prior to term, a human fetus also practices sucking, swallowing and even rudimentary breathing actions [93]. These actions are sometimes collectively described as ‘motor babbling’, providing movement feedback for the fetus that allows its brain to understand the concept of space and the consequence of its own actions [91]. Even at this prenatal stage, the hypothalamus may play a role in coordinating behaviours through early life hypothalamic projections to the motor circuits in the brain stem and spinal cord [42,94].

### (b) Mammalian social behaviours

Other self-initiated behaviours are manifest in the early postnatal period and serve to shape higher-order aspects of brain function, including social cognition. Because actions in the postnatal period are often concerned with immediate survival, the spontaneous behaviours are generally viewed within that context. However, early behavioural experiences related to nutrition, protection and threat avoidance also provide valuable experiences for learning, exposing the developing brain to a range of complex stimuli and social interactions that have the additional role of shaping its sensory, motor and cognitive circuits.

In mammals, a shared behaviour is the suckling action of a newborn from its mother. While the manifestation of this behaviour varies across diverse species with different litter structures and levels of altriciality, its core aspects are conserved. Newborn suckling behaviour depends critically on circuits in both the hypothalamus, to instigate behaviour, and olfactory structures responsive to pheromones released by the mother [95–97]. This internal drive and innate sensory capacity are critical for survival, as newborn mammals that do not suckle successfully from their mothers cannot survive [98]. At the same time, the act of suckling initiates more complex behaviours, including interactions related to long-term social bonding. For example, the attractant pheromones expressed by the mother to encourage suckling, together with the olfactory signals learned during this period, support the mutual recognition between newborns and mothers, as well as newborn recognition of siblings [99,100]. This recognition and the early life interactions prior to weaning, initiate longer-term dyadic relationships with affiliative and competitive behaviours, and even eventual mating decisions [101]. Following weaning, these relationships change [102] and the brain must be sufficiently trained to undertake new experiences such as obtaining food and confronting predators. This set of stages is, in some sense, genetically programmed and built into the maturation of the mammalian brain.

### (c) Primate social behaviours

While olfaction is a dominant social modality among mammals, particularly in early life [103], vision is also important, particularly in large mammalian species such as primates and ungulates [104]. Vision affords animals with the capacity to quietly observe their environment, including conspecifics, prey and predators, from a distance. It also serves to mediate important physical interactions with the environment, such as the placement of the forelimb during locomotion and visual guidance of manual reaching and grasping. In the social domain, vision provides a medium for recognizing and interpreting other individuals. In the primate brain, multiple cortical areas appear specialized for the perception of faces, bodies and other social information [105–107], with evidence that some such features are shared among mammals [104,108,109]. Studies in humans and monkeys suggest that face expertise and the cortical areas that support it emerge during infancy and mature gradually [52,110–114]. Importantly, this maturation requires experience with faces during this developmental period. For example, while the location of cortical face patches appears genetically predetermined, they exhibit their normal adult functionality only following experience with faces [6,115–117].

Thus, the primate brain is obligated to create the conditions for gaining experience with faces in order to reach its full potential in domains such as individual recognition and the reading of facial expressions. These conditions are, in part, set in place by early life social behaviours that might drive exposure to faces. Social and exploratory play is an important element for brain development that is common to most animal species and most strongly expressed during infancy [118]. Moreover, comparative analysis across primates suggests that the amount of social play is linked, in part, to the actions of the hypothalamus [119]. Some evidence suggests that the medial prefrontal cortex, perhaps through its substantial interaction with the hypothalamus [83], may play an explicitly prosocial role in directing attention toward social stimuli early in life [6,120]. Also important are innate stimulus biases toward faces that begin to shape the expression of this social behaviour almost immediately after birth [121–123]. One hypothesis suggests that early attention to faces is imposed by genetically specified sensorimotor circuitry within the superior colliculus [7,124]. Some support for this hypothesis comes from the identification of very short-latency neurons in the superior colliculus of the adult macaque that respond selectively to face-like patterns [125–127]. As the telencephalon gains experience with the species-typical range of social interactions, including the rules of looking behaviour in dyadic interactions and normal variation in facial structure andbehaviour, it increasingly takes over the direction of most aspects of social exchange.

One important and difficult question is how the brain, through extensive experience with complex stimuli such as individual faces, modifies itself through its accumulated experiences. Recent work studying the encoding of faces suggests that an important mode of learning in the cerebral cortex involves the utilization of learned norms [128]. At an abstract level, norms are internally stored references that the brain has extracted through experience [129,130]. In the case of faces, the brain represents faces relative to an internally stored template of average facial structure that is presumably learned through experience [131–137]. It is interesting to speculate that normative learning is a fundamental and evolutionarily conserved principle that guides plasticity in the telencephalon. According to this idea, self-tuition leads to the gradual extraction and internalization of norms in multiple domains, honing an animal's behavioural repertoire, and make it more sensitive to subtle stimuli and events. This mode of learning creates a predictive framework, which carries great advantage for cognitive performance [138–141], as well as survival and procreation.

## 5. Conclusion

In this article, we discussed the prospect that the basic design of the vertebrate brain has evolved to meet the needs of developmental self-tuition. This conserved paradigm for achieving adult-level brain function uses behaviours spurred by motivated states originating in the hypothalamus, along with innate stimulus preferences built into sensory circuits, to compel certain species-appropriate modes of interaction with the environment. These interactions gradually train the non-hypothalamic telencephalon to take over important aspects of perception and behaviour, including the direction of its own education. This learning benefits from the gradual statistical embedding of important information about the world, affording the telencephalon intelligence and autonomy over multiple domains of cognition. Only through such learning are vast regions of the brain able to achieve their adult functions. As such, programmed self-tuition is an essential developmental paradigm, facilitating competitive survival and procreation in an unbroken chain of vertebrate evolution.

## Data accessibility

This article has no additional data.
